# ABS Nanocomposites for Advanced Technical and Biomedical Applications

**DOI:** 10.3390/polym17070909

**Published:** 2025-03-27

**Authors:** Lubomír Lapčík, Martin Vašina, Yousef Murtaja, Harun Sepetcioglu, Barbora Lapčíková, Martin Ovsík, Michal Staněk, İdris Karagöz, Apurva Shahaji Vadanagekar

**Affiliations:** 1 Department of Physical Chemistry, Faculty of Science, Palacky University, 17. Listopadu 12, 771 46 Olomouc, Czech Republic; barbora.lapcikova@upol.cz (B.L.); apurva.vadanagekar01@upol.cz (A.S.V.); 2 Faculty of Technology, Tomas Bata University in Zlin, Vavreckova 5669, 760 01 Zlin, Czech Republic; martin.vasina@vsb.cz (M.V.); murtaja@utb.cz (Y.M.); ovsik@utb.cz (M.O.); stanek@utb.cz (M.S.); 3 Department of Hydromechanics, Faculty of Mechanical Engineering, VŠB—Technical University of Ostrava, and Hydraulic Equipment, 17. Listopadu 15/2172, 708 33 Ostrava-Poruba, Czech Republic; 4 Department of Metallurgy and Materials Engineering, Technology Faculty, Selçuk University, 42075 Konya, Türkiye; harunsepet@selcuk.edu.tr; 5 Department of Polymer Materials Engineering, Faculty of Engineering, Yalova University, 77200 Yalova, Türkiye; idris.karagoz@yalova.edu.tr

**Keywords:** nanocellulose, halloysite, calcium carbonate, ABS polymer, mechanical testing

## Abstract

This study investigated the mechanical, thermal, and morphological properties of acrylonitrile butadiene styrene (ABS)-based nanocomposites reinforced with different types and concentrations of nanofillers. The uniaxial tensile testing results indicated that Young’s modulus (*E*) generally decreased with increasing filler content, except at 0.500 w.% filler concentration, where a slight increase in stiffness was observed. A statistically significant interaction between sample type and filler concentration was identified (*p* = 0.045). Fracture toughness measurements revealed a significant reduction in impact resistance at 1.000 w.% filler concentration, with values dropping by up to 67% compared with neat acrylonitrile butadiene styrene. Dynamic mechanical vibration testing confirmed a decrease in stiffness, as evidenced by a shift of the first resonance frequency (*f_R_*_1_) to lower values. Hardness measurements including indentation and Shore D hardness exhibited an increasing trend with rising filler concentration, with statistically significant differences observed at specific concentration levels (*p* < 0.05). Scanning electron microscopy analysis showed that nanofillers were well dispersed at lower concentrations, but agglomeration began above 0.500 w.%, resulting in void formation and a noticeable decline in mechanical properties. The results suggest that an optimal filler concentration range of 0.250–0.500 w.% offers an ideal balance between enhanced mechanical properties and material integrity.

## 1. Introduction

Nanocomposites, especially those based on calcium carbonate (CaCo_3_) [1,2] and halloysite [3], have been a subject of scientific research for a long time. Hollow spheres, due to their uniform shapes, provide consistent properties independent of orientation. Nano- and micro-sized hollow spheres, synthesized by various methods [4,5,6], act as colloidal particles, offering the advantage of moving freely and preventing entanglement in complex formulations [6].

Nano-calcium carbonate (nCaCO₃) is highly effective in enhancing the mechanical properties of polymer composites, especially hardness and impact resistance [2]. However, it has moderate heat resistance. Halloysite nanotubes (HNTs), with their unique tubular morphology, offer excellent reinforcement properties [3] and the potential for controlled-release applications. Due to their exceptional tensile strength, biodegradability, and antimicrobial properties [7], nanocellulose fibers (NCFs) are considered ideal candidates for sustainable applications requiring lightweight but high-strength materials.

In recent years, there has been increasing interest in using nanocellulose as a reinforcing material in composites. Moreover, it is recognized as an important substrate for the synthesis of metallic nanoparticles due to its unique porous structure, biodegradability, and biocompatibility. It provides a controlled environment for crystal growth and nucleation, enabling precise control over particle size and dispersion stability. Nanocellulose offers an environmentally friendly alternative for nanoparticle synthesis without the need for external reducing agents [8].

Nanocellulose can be obtained through top-down approaches, such as the enzymatic or mechanical processing of plant fibers, or biosynthesis by bacteria. There are three main types of nanocellulose, microfibrillated cellulose (MFC), nanocrystalline cellulose (NCC), and bacterial nanocellulose (BNC), which differ in structure, production techniques, and properties. Due to their high surface area, hydrophilicity, and biocompatibility, nanocellulose composites are widely used in medical devices and eco-friendly materials [9].

Nanocellulose has been identified as a sustainable material for food packaging applications due to its enhanced mechanical strength, biodegradability, and microbial resistance [10]. Additionally, smart packaging technologies such as freshness monitoring with pH-sensitive dyes have been employed. Improvements in mechanical and thermal properties have been reported in composites containing nanocellulose [11]. In medical applications, it has also proven beneficial for wound coatings and drug delivery systems [12]. Furthermore, nanocellulose has been used in water-repellent (hydrophobic) fabrics, durable coatings [13], and environmental sensors [14], highlighting its multifunctional industrial potential.

Early research focused on the use of cellulose as a reinforcing material in polymers, but recent developments have expanded to the use of nanocellulose as a high-performance filler in various composites. The first studies in 1987 explored the feasibility of cellulose as a reinforcing material in polymers like polypropylene (PP), polystyrene (PS), and high-density polyethylene (HDPE). These early studies led to significant advancements in the development of nanocomposite materials. Subsequent research has generally focused on the use of nanocellulose as a reinforcing filler in various synthetic polymers [15].

Despite extensive research on nanocrystalline cellulose (CNC)-based ABS nanocomposites, the effects of cellulose nanofibers (CNFs) in these systems remain largely unexplored. Most studies have focused on cellulose nanocrystals (CNCs), while the high surface area, network-forming capability, and unique properties of CNFs in ABS composites have not been fully explored. This gap presents an opportunity to investigate the integration of CNFs as reinforcements in ABS matrices. This could lead to significant advances in the development of sustainable, high-performance nanocomposites. Later studies have shown that the formation of a percolation network at low concentrations significantly enhances the mechanical properties of the composite materials. The surface properties and versatility of nanocellulose allow modifications in mechanical properties such as hardness and flexibility, making it suitable for a wide range of applications [11].

The integration of cellulose nanofibers (CNFs) into the ABS matrix occurs through hydrogen bonds and physical interactions between the CNFs and polymer chains. These interactions enhance the distribution of fibers within the matrix. The high surface area and functional groups of CNFs form strong interactions with the ABS polymer, leading to mechanical locking and more efficient load transfer. These interactions are expected to improve the overall mechanical properties, such as tensile strength, impact resistance, and thermal stability. Additionally, the percolation network formed by CNFs in the ABS matrix could enhance water absorption resistance and dimensional stability.

Research on the reinforcement of ABS with cellulose nanocrystals (CNCs) extracted from sugarcane bagasse has shown significant changes in the mechanical and thermal properties of ABS. An optimal CNC concentration range of approximately 0.5 to 0.7 w.% increased the impact strength, elastic modulus, and storage modulus in the ABS. However, exceeding this concentration led to the agglomeration of CNCs and poor dispersion in the ABS matrix, resulting in a decrease in tensile strength and thermal stability. Thus, balancing between property enhancement and overloading has been recognized as a critical factor in the development of high-performance ABS/CNC nanocomposites [16].

A study that investigated the effects of modified cellulose nanocrystals on ABS nanocomposites reported improvements in the elastic modulus and yield strength at a 1 w.% filler concentration. However, the increase in hardness led to a decrease in impact strength [17]. Furthermore, it was observed that cellulose nanocrystals did not significantly affect the glass transition temperature of the ABS matrix [18].

Feng et al. [19] explored the reinforcement of 3D-printed ABS using a mixture of methacrylate resin and cellulose nanocrystals (CNCs). The study found that treating the 3D-printed ABS with this mixture significantly improved its mechanical properties, especially at an 80% filler density. Thermal stability also increased, with improvements in parameters such as glass transition temperature (*T_g_*) and maximum weight loss rate (*T_max_*). Additionally, the addition of CNC improved the uniformity of the printed material [20]. CNCs have been extensively employed in the manufacturing of nanocomposites, particularly those based on ABS, according to the current understanding of the authors [19]. At present, potential therapeutic applications of prototyped ABS devices have been studied in vivo and in vitro [21,22].

Although most research to date has focused on CNC-based ABS nanocomposites, there is limited work on the potential of cellulose nanofibers (CNFs) in these systems. However, there is a lack of comprehensive studies on the use of CNFs as reinforcements in ABS nanocomposites. To fill this gap, the present study aims to investigate the effects of CNFs on ABS nanocomposites through a comparative analysis. The goal is to gain new insights into the interactions of CNFs with the polymer matrix. By examining the differences in CNF concentrations and processing techniques, optimal strategies for achieving superior mechanical, thermal, and barrier properties in ABS composites will be identified. The primary aim of this research is to evaluate the improvements in thermal, mechanical, and water absorption properties due to the integration of bio-based reinforcements into the ABS matrix and to comprehensively investigate the reinforcing effectiveness of CNFs. This research aims to deepen the understanding of the potential applications of bio-based composites in industries such as automotive, electronics, packaging, construction, and sports equipment. In this context, the mechanical and dynamic properties obtained (such as increased Young’s modulus, yield stress, crack resistance achieved with the use of optimal nanofillers, and improvements in surface and microhardness) can be directly related to relevant application requirements. For example, in the automotive industry, benefits such as high hardness and vibration damping; in electronics, resistance to scratching and impact damage; in packaging, flexibility and impact absorption; in construction, load-bearing capacity and resistance to shape deformation; and in sports equipment, light weight and impact absorption are advantages provided by the use of nanofillers at optimal concentrations (particularly in the range of 0.250–0.500 w.%). In this way, the correlation of material properties with industrial applications enables the development of sustainable and high-performance composite designs.

## 2. Materials and Methods

### 2.1. Materials

In this study, Nano-calcium carbonate (NCC), halloysite nanotubes (HNTs), and cellulose nanofibers (CNFs) were used to modify virgin ABS. For the preparation of ABS nanocomposites, a commercial grade ABS 750SW (Kumho Petrochemical, Seoul, Republic of Korea) was used as the matrix material. It had a density of 1.04 g/cm^3^ and a Melt Flow Index (MFI) of 38 g/10 min. The NCC filler was supplied by Adacal Co. (Konya, Türkiye) and was treated with stearic acid prior to the further processing. The average particle size (*d*_50_) of 0.05 µm was obtained from SEM measurements. The chemical compositions and physical properties of the NCC used are available elsewhere [19,23]. The virgin ABS reinforced with HNTs (Al_2_Si_2_O_5_(OH)_4_), featuring a two-layered nanocylindrical structure, had an inner diameter ranging from 1 to 20 nm, an outer diameter of 30–50 nm, and a length of 100–800 nm (supplied by Esan Eczacıbaşı Company, Istanbul, Türkiye). Additionally, virgin ABS was modified with CNFs, which had a diameter of 10–20 nm, a length of 2–3 µm, a density of 1.50 g/cm^3^, and a crystallinity ratio of 92% (purchased from Nanografi Company, Ankara, Türkiye).

#### Preparation of ABS Nanocomposites and Test Samples

ABS and nanofillers (NCCs, HNTs, and CNFs) were initially placed in a bucket and subjected to mechanical blending for 10 min. After blending, the material was extruded into filament form using a Robotdigg SJ25 extruder with a specialized mixing screw, employing the melt-blending method. The temperature profile during the extrusion was maintained at 25/60/160/180 °C. The extruder had a screw diameter of 25 mm, a screw speed of 1300 RPM, and a torque of 20 Nm. The screw’s length-to-diameter (*L*/*D*) ratio was 16. The ABS/NCCs, ABS/HNTs, and ABS/CNFs nanocomposite filaments were then granulated using a granulator. The granulated nanocomposites were dried at 80 °C for 2 h in a Binder brand oven to remove moisture before the injection-molding process. Test specimens were produced via injection molding from the granulated mixtures using the Spex 400 model injection machine (Engel Austria, Steyr, Austria), in compliance with ISO 294 standards. The molding parameters included an injection pressure of 80 bar, an injection speed of 50 mm/s, a holding pressure of 55 bar, and a cycle time of 30 s. Five sample groups were prepared: pure ABS and ABS nanocomposites reinforced with nanofillers at weight fractions of 0.125%, 0.250%, 0.500%, and 1.000%. These samples were produced under identical conditions to ensure consistency and reliable comparisons. Each nanofiller type was systematically incorporated into the ABS matrix at the specified concentrations, providing a structured approach for evaluating the influence of the nanofiller type and its concentration on the mechanical properties of the studied nanocomposites. The test samples were systematically labeled: Sample 1 (ABS/NCCs), Sample 2 (ABS/HNTs), and Sample 3 (ABS/CNFs). This labeling facilitated clear differentiation for subsequent analysis [24,25].

### 2.2. Methods

#### 2.2.1. Uniaxial Tensile Testing

Tensile testing was conducted using a Zwick/Roell Vibrophore 100 (Zwick Roell, Ulm, Germany), in accordance with the ČSN EN ISO 527-1 standard. The test samples had dimensions of 170 mm × 20 mm × 2 mm (length × width × thickness). Measurements were taken at room temperature (25 °C) with a strain rate of 10 mm/min. Young’s modulus of elasticity (*E*), yield stress, and elongation at maximum tensile force (*dL_max_*) were determined from stress–strain plots. Each experiment was repeated three times, and the mean values with standard deviations were calculated.

#### 2.2.2. Charpy Pendulum Impact Testing

Impact tests were performed using a Zwick/Roell HIT25P machine (Zwick Roell, Ulm, Germany) in accordance with the ČSN EN ISO 179-2 standard, with an energy drop of 7.5 J. The test samples had dimensions of 100 mm × 10 mm × 2 mm. Each experiment was repeated three times, and the mean values and standard deviations of the fracture toughness (*a_k_*) were calculated. All tests were conducted at 25 °C.

#### 2.2.3. Displacement Transmissibility Measurements

The dynamic mechanical properties of the specimens under harmonic loading were evaluated using the displacement transmissibility (*T_d_*), which is defined as follows [26]:

(1) $$T_{d}=\frac{y_{2}}{y_{1}}=\frac{a_{2}}{a_{1}},$$

where *y*_1_ is the displacement amplitude on the input side, *y*_2_ is the displacement amplitude on the output side, and *a*_1_ and *a*_2_ are the corresponding acceleration amplitudes. The displacement transmissibility of a spring-mass-damper system is defined by

(2) $$T_{d}=\sqrt{\frac{k^{2}+{\left(c\cdot \omega \right)}^{2}}{{\left(k-m\;\cdot \;\omega^{2}\right)}^{2}+{\left(c\;\cdot \;\omega \right)}^{2}}}=\sqrt{\frac{1+{\left(2\;\cdot \zeta \cdot r\right)}^{2}}{{\left(1-r^{2}\right)}^{2}+{\left(2\;\cdot \;\zeta \cdot r\right)}^{2}}}$$

where *ω* is the angular frequency, ζ is the damping ratio, and *r* is the frequency ratio. Mechanical vibration tests were conducted using the forced oscillation method, and displacement transmissibility was measured with a BK 4810 vibrator, BK 3560-B-030 signal pulse multi-analyzer, and a BK 2706 power amplifier over a frequency range of 2 to 3200 Hz. The test specimens were fitted with BK 4393 accelerometers (Brüel & Kjær, Nærum, Denmark) to record acceleration amplitudes. The measurements were conducted at ambient conditions (25 °C), with each measurement repeated four times.

#### 2.2.4. Microhardness Testing

Micro-indentation tests were performed using a Micro Combi Tester (Anton Paar, Graz, Austria) following the ČSN EN ISO 14577 standard. A cube-corner diamond tip (Vickers, Anton Paar, Graz, Austria) was used with a maximum load of 3 N, a loading/unloading rate of 6 N/min, and a holding time of 90 s. The test samples measured 100 mm × 10 mm × 2 m. The indentation modulus (*E_IT_*) was calculated using the plane strain modulus of elasticity (E*) and an estimated Poisson’s ratio (*ν*) of 0.35 for the samples [27,28]:

(3) $$E_{IT}=E^{*}\left(1-\nu^{2}\right)$$

Indentation hardness (*H_IT_*) was also measured. Each test was repeated three times, and the mean values with standard deviations were calculated. All tests were conducted at room temperature (25 °C).

#### 2.2.5. Shore D Hardness Measurements

Shore D hardness measurements were conducted using an AFFRI Microhardness DM2D instrument (AFFRI, Inc., Wood Dale, IL, USA), with a 4 s holding time using the Shore D method. The test samples were 100 mm × 10 mm × 2 mm. Each measurement was repeated three times, and the mean values and standard deviations of the Shore D hardness were calculated. All tests were performed at room temperature (25 °C).

#### 2.2.6. Statistical Analysis

Experimental results were expressed as mean values with standard deviations. Statistical analysis was conducted using SigmaPlot 12.5 software (Systat Software, Inc., San Jose, CA, USA). Significance was evaluated using two-way ANOVA with a significance level of *p* < 0.05. Data normality was confirmed using the Shapiro–Wilk test.

#### 2.2.7. Particle Distribution and Agglomeration Analysis Based on SEM Images

The scanning electron microscope (SEM) images were analyzed to examine particle distribution and agglomeration in the samples. Fractured surfaces were analyzed using a scanning electron microscope EVO LS10 (Carl Zeiss Microscopy GmbH, Jena, Germany). Prior to imaging, the samples were coated with a 100 Å-thick gold layer to improve conductivity and prevent oxidation. Microstructural images were acquired at an acceleration voltage of 20 kV. The SEM images were analyzed using Python-based image processing techniques (Python Software Version 3.11.2). After converting images to grayscale, the Canny filter was applied for edge detection, and connected component analysis (CCA) was used to determine particle count and size distribution. A Gaussian-based density map was generated to assess spatial particle distribution, providing a detailed evaluation of agglomeration tendencies.

## 3. Results and Discussion

### 3.1. Uniaxial Tensile Testing

**Figure 1:**
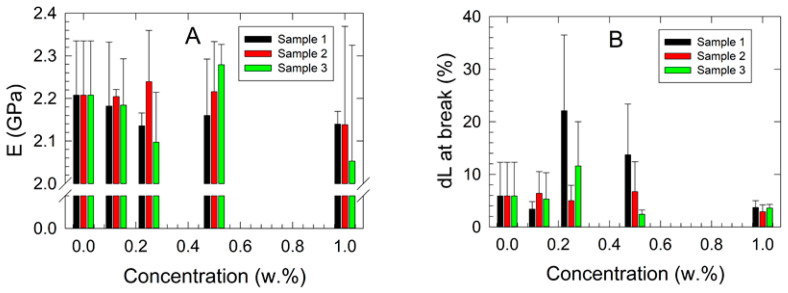
Results of the uniaxial tensile testing of studied ABS nanocomposites: (**A**) Young’s modulus of elasticity vs. filler concentration, (**B**) elongation at break vs. filler concentration. Deformation rate 10 mm/min.

The results of the mechanical tensile testing of the studied samples are presented in Figure 1. Typical force vs. stroke patterns for 1.000 w.% filler concentration in the studied nanocomposites and neat ABS matrix are shown in the Supplementary Materials, Figure S1. It was observed that, up to a concentration of 1.000 w.% for Samples 1 and 3, the corresponding Young’s modulus of elasticity (*E*) exhibited a decreasing trend (Figure 1A), except for the samples containing 0.500 w.% filler concentration. At this concentration, an increase in mechanical stiffness was observed for both samples, as indicated by the increasing *E*. However, an opposite trend was noted for Sample 2, where an increased *E* was detected at concentrations below 0.500 w.%, followed by a significant decrease in mechanical stiffness at the 1.000 w.% filler concentration. It was assumed that the anomalous behavior of Young’s modulus at the 0.500 w.% filler concentration resulted from inhomogeneous dispersion, weak interfacial interactions, or filler agglomeration, which reduced reinforcement efficiency. Additionally, slight plasticization effects and experimental variability may have contributed to this anomaly.

The observed results corresponded well with the increasing elongation at break (*dL*) in the low concentration range (below 0.250 w.%), indicating enhanced ductility of the samples. Conversely, at the highest filler concentration of 1.000 w.%, the opposite behavior was observed for all studied nanocomposites, characterized by a 50 % significant decrease in *dL* at break compared with the virgin ABS (Figure 1B).

The two-way ANOVA results for the *E* data sets indicated that concentration has a statistically significant effect on the dependent variable (*p* = 0.012). However, no significant differences were observed among the samples as a whole (*p* = 0.512). Notably, a statistically significant interaction between concentration and sample was detected (*p* = 0.045), suggesting that the effect of concentration is sample-dependent. These findings emphasize the necessity of considering both main and interaction effects when evaluating the impact of concentration on the measured outcomes.

**Figure 2:**
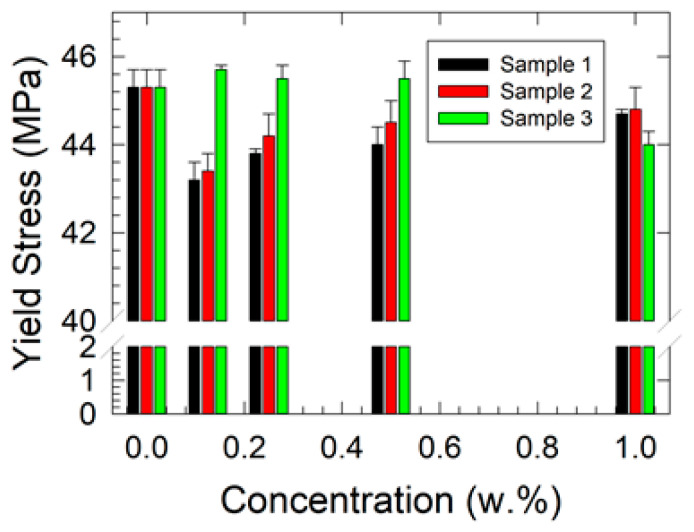
Results of the yield stress vs. filler concentration dependencies as obtained by uniaxial tensile testing at the 10 mm/min deformation rate.

Figure 2 shows the relationship between yield stress and nanofiller concentrations. The results suggest that nanoparticle fillers may act as a limiting factor in the polymer’s ability to undergo plastic deformation under mechanical load in Samples 1 and 2. This effect could lead to a lower yield strength of the composites, as the material absorbs less energy before reaching the yield point. However, in Sample 3, the opposite reinforcing effect was observed. It is assumed that when the nanocellulose fibrous filler is evenly dispersed and exhibits good compatibility with the polymer matrix, a higher concentration contributes to composite reinforcement and an increase in yield strength, as clearly demonstrated in Figure 2.

The results of the two-way ANOVA for the yield stress data indicated that concentration does not have a statistically significant effect on the investigated composite types (*p* = 0.158) at the *p* = 0.05 level. In contrast, while the effect of sample type showed a trend toward significance (*p* = 0.086), it did not reach the conventional threshold for statistical significance. Furthermore, no significant interaction effect between concentration and sample type was observed (*p* = 0.919). These findings suggest that neither concentration nor its interaction with sample type have a meaningful impact on the measured yield stress.

However, it should be noted that an optimal amount of nanoparticle filler is critical, as excessive concentrations may lead to the agglomeration of nanofibers and a reduction in mechanical properties, as observed for the 1 w.% nanocellulose filler concentration.

### 3.2. Fracture Toughness Measurements

**Figure 3:**
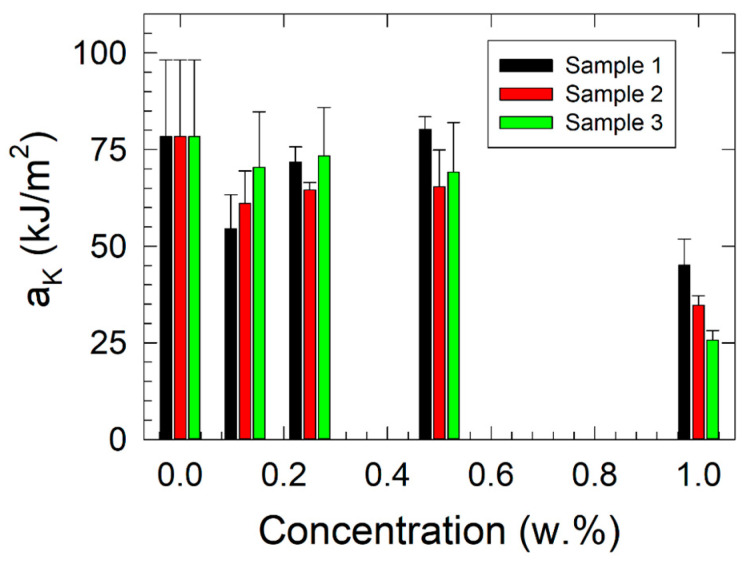
Results of the fracture toughness vs. filler concentration measurements as obtained by Charpy pendulum measurements.

The results of fracture toughness measurements are shown in Figure 3. A decrease in the fracture toughness (*a_K_*) of the polymer composite was observed with increasing filler concentration due to the composite’s higher brittleness and the limited mobility of the matrix polymer chains. The formation of defects due to the agglomeration of filler particles resulted in a reduction in plastic deformation of the matrix. Therefore, based on the obtained results, an optimal filler content for achieving a good balance between the mechanical stiffness, material strength, and fracture toughness of the investigated nanocomposites appears to be in the 0.250 to 0.500 w.% filler concentration range. A substantial decrease in fracture toughness was observed for nanocomposites with a filler concentration of 1.000 w.% of all studied fillers compared with the primary ABS matrix, with values dropping from the original 78.34 kJ/m^2^ to 45.06 kJ/m^2^ (Sample 1), 34.65 kJ/m^2^ (Sample 2), and 25.7 kJ/m^2^ (Sample 3). The *F*-value for concentration (14.83) exceeded the threshold (*F_critical_* = 3.48), indicating that the differences between concentrations are statistically significant. In contrast, the *F*-value for sample types (2.45) was lower than the critical value, suggesting that the differences between samples are not statistically significant.

### 3.3. Vibration-Excited Dynamic Mechanical Testing

**Figure 4:**
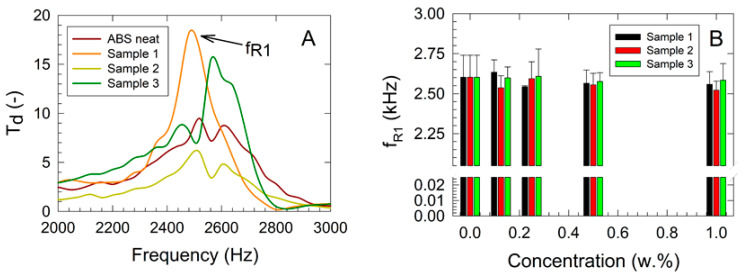
Results of the dynamic mechanical vibration damping measurements of studied composites: (**A**) frequency dependencies of the displacement transmissibility, (**B**) first resonance frequency vs. filler concentration data.

The results of the dynamic mechanical vibration testing are presented in Figure 4. A shift of *f_R_*_1_ to lower frequencies with increasing filler concentration was observed, indicating a decrease in the mechanical stiffness of the studied nanocomposites [29]. This trend was consistent across all samples. As shown in Figure 4B, the highest mechanical stiffness was recorded for the nanocellulose fiber composites (Sample 3), as reflected in the higher *f_R_*_1_ values compared with other composites, regardless of filler concentration. For the 1.000 w.% filler concentration, the shift of *f_R_*_1_ to lower frequencies ranged from 0.69% for Sample 3 to 3.09% for Sample 2 and 1.68% for Sample 1 compared with the virgin ABS sample. A two-way ANOVA was performed, revealing no statistically significant differences in mean values for either filler concentration (*p* = 0.359) or sample type (*p* = 0.273), suggesting that any observed differences were likely due to random sampling variability.

### 3.4. Microhardness and Shore D Hardness Testing

**Figure 5:**
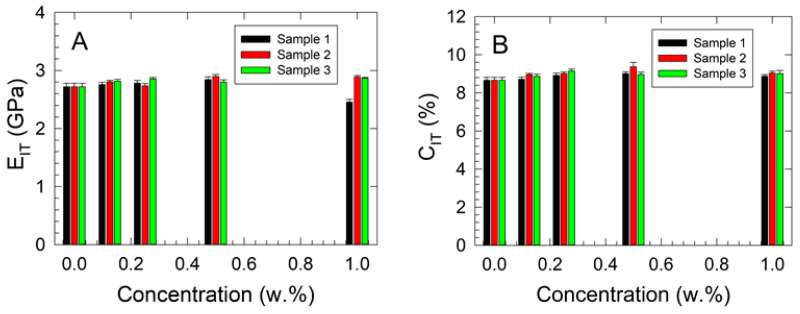
Results of the microhardness measurements: (**A**) indentation modulus vs. filler concentration, (**B**) indentation creep vs. filler concentration dependencies.

**Figure 6:**
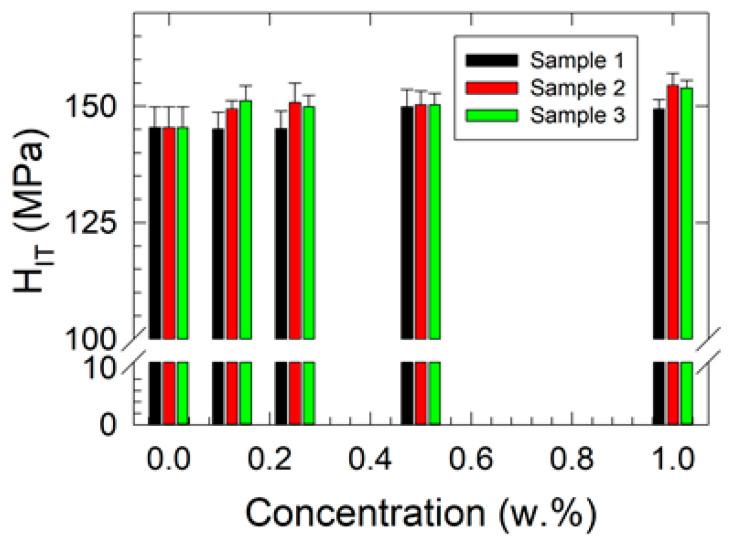
Concentration dependencies of the indentation hardness of the studied nanocomposites.

**Figure 7:**
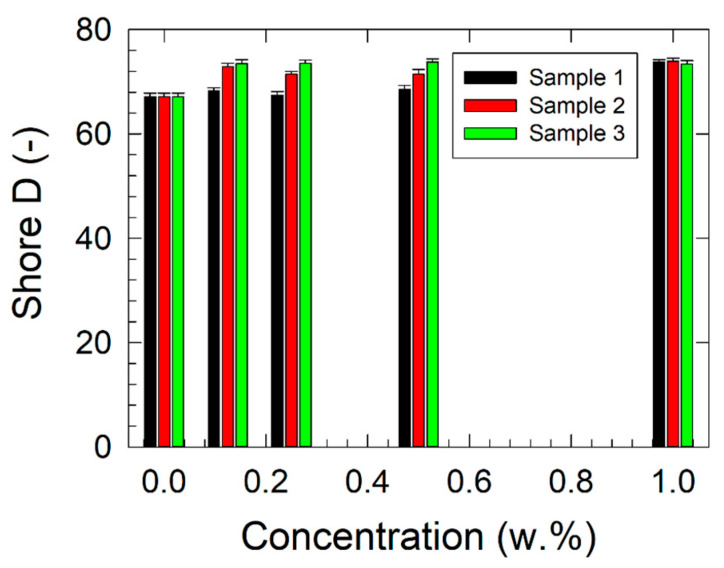
Concentration dependencies of the Shore D hardness of the studied nanocomposites.

The results of the microhardness measurements are presented in Figure 5 and Figure 6; those of Shore D hardness are shown in Figure 7.

An increasing trend in *E_IT_* with rising filler concentrations of approximately 5.1% was observed as a general pattern for a 1.000 w.% concentration in all studied samples (Figure 5A), except for Sample 1, where a significant decrease of around 10% was noted compared with the neat ABS sample. The results of the two-way ANOVA for *E_IT_* indicated that the assumptions of normality and equal variance were met. No statistically significant difference was observed between concentration levels (*p* = 0.665) or between samples (*p* = 0.327), suggesting that variations in mean *E_IT_* values were due to random sampling variability. The mean *E_IT_* values (in GPa) for the different concentration levels (in w.%) were 0.000 (2.721), 0.125 (2.792), 0.250 (2.792), 0.500 (2.847), and 1.000 (2.735). The values for the individual samples were Sample 1 (2.712), Sample 2 (2.809), and Sample 3 (2.811). The power of the test was found to be low, particularly for concentration (0.0502) and samples (0.0809), at a significance level of *p* = 0.05.

The indentation creep results are summarized in Figure 5B. A steady increase in indentation creep (*C_IT_*) with rising filler concentrations was observed, indicating increased interfacial plasticity. The results of the two-way ANOVA for *C_IT_* confirmed that the assumptions of normality and equal variance were met. A statistically significant relationship was identified between concentration levels and *C_IT_* values (*p* = 0.007), whereas no significant difference was found between the samples (*p* = 0.096). The mean *C_IT_* values (in %) for different concentration levels (in w.%) were 0.000 (8.659), 0.125 (8.848), 0.250 (9.028), 0.500 (9.117), and 1.000 (8.977). The values for individual samples were Sample 1 (8.835), Sample 2 (9.008), and Sample 3 (8.934). Statistically significant differences between certain concentration levels were confirmed by the Tukey test. The power of the test was determined to be high for concentration (0.907) but low for samples (0.315) at a significance level of *p* = 0.05.

The indentation hardness results are presented in Figure 6. An increasing trend in *H_IT_* with rising filler concentration was observed. The results of the two-way ANOVA for *H_IT_* confirmed that the assumptions of normality and equal variance were met. A statistically significant difference was identified between concentration levels (*p* = 0.007), indicating that variations in mean *H_IT_* values could not be attributed to random sampling variability. A statistically significant difference was also detected between the samples (*p* = 0.024), suggesting that the differences in mean *H_IT_* values among samples were not due to random variability. The mean *H_IT_* values (in MPa) for different concentration levels (in w.%) are reported as follows: 0.000 (145.437), 0.125 (148.551), 0.250 (148.628), 0.500 (150.141), and 1.000 (152.591). The corresponding values for the individual samples were Sample 1 (146.990), Sample 2 (150.066), and Sample 3 (150.153). The power of the test was found to be high for concentration (0.892) and moderate for samples (0.655) at a significance level of *p* = 0.05. Statistically significant differences between certain concentration levels were confirmed by the Tukey test, specifically, between 1.000 w.% and 0.000 w.% (*p* = 0.004) and between 0.500 w.% and 0.000 w.% (*p* = 0.044). Additionally, significant differences were observed between samples, particularly between Sample 3 and Sample 1 (*p* = 0.036) and between Sample 2 and Sample 1 (*p* = 0.041).

A similar increasing trend in Shore D hardness with rising filler concentration was observed, as shown in Figure 7. The highest increase in Shore D hardness, approximately 9.4% at 1.000 w.%, was recorded for all studied samples compared with virgin ABS. At concentrations ranging from 0.125 to 0.500 w.%, the highest Shore D hardness was observed for the nanocellulose composites (Sample 3), followed by Sample 2 and Sample 1.

### 3.5. SEM Analysis of Particle Distribution and Agglomeration

**Figure 8:**
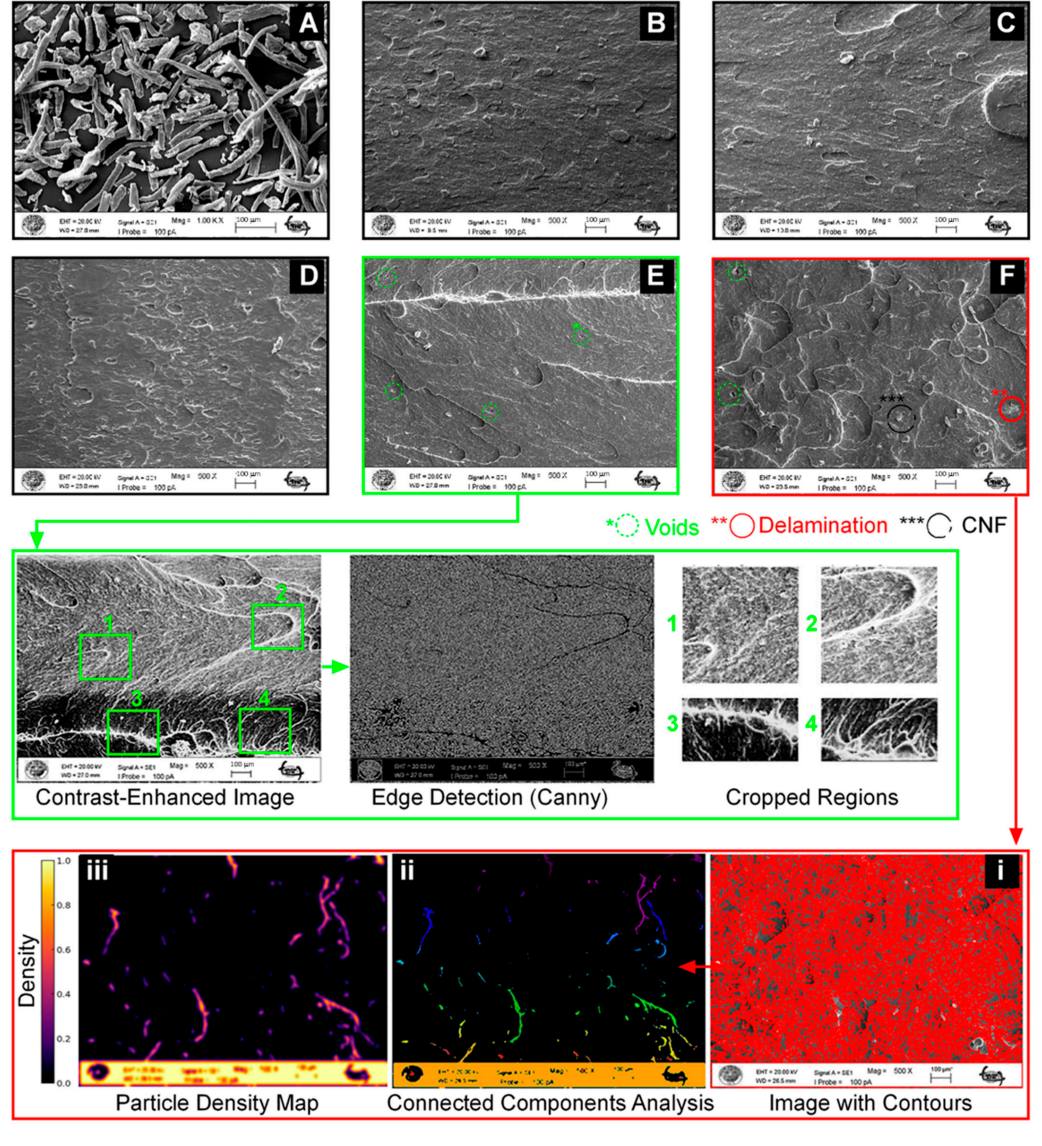
SEM microstructure images: (**A**) cellulose nanofibers, (**B**) Pure ABS, (**C**) 0.125 w.% CNFs filler concentration, (**D**) 0.250 w.% CNFs filler concentration, (**E**) 0.500 w.% CNFs filler concentration, (**F**) 1.000 w.% CNFs filler concentration.

The SEM images of the fracture surfaces of the samples (Figure 8) revealed that the pure ABS sample exhibits a smooth fracture surface. With the addition of CNFs, a slight increase in surface roughness was observed in the composite fracture surfaces. The SEM images demonstrate that CNFs are homogeneously distributed within the ABS matrix to a certain extent. However, as the CNF content increases, the tendency for agglomeration also rises. The formation of long and thin fibrils provides a large surface area, and due to the high density of hydroxyl groups on the fibril surface, strong interactions between the fiber bundles further enhance the agglomeration tendency [30]. Additionally, the presence of voids in certain regions may indicate weak interfacial adhesion between the filler and the matrix, largely attributed to agglomeration [31].

The specific regions in Figure 8E, where dense white areas (1,2) and irregular clusters (3,4) are visible, mark the onset of agglomeration. As the nanofiller content increases, numerical analysis of SEM images (Figure 8F(i–iii)) confirms the growing presence of agglomeration. According to CCA results, a high degree of variability in particle sizes was observed. The average particle size was determined to be 552 pixels, while the median size was 20 pixels. The high standard deviation (5799 pixels) suggests that while many particles were relatively small, some clusters were significantly larger, further confirming the presence of agglomeration.

To further analyze the spatial distribution of particles, a Gaussian-based density map was generated. This analysis confirmed that the particles were not homogeneously distributed throughout the sample, with localized clusters exhibiting high particle density in specific regions. These high-density regions may have formed due to insufficient dispersion or strong interparticle interactions resulting from an increased concentration ratio within the structure.

Agglomeration negatively affected the mechanical properties of the material (Figure 2). As observed in Figure 8, particle clustering can lead to stress concentration points, potentially weakening the structural integrity and overall performance of the material. The obtained results (Figure 2) further support this phenomenon.

## 4. Conclusions

This study found that nanofiller concentration significantly affected Young’s modulus of elasticity (*p* = 0.012) with a statistically significant concentration–sample interaction (*p* = 0.045). Specifically, Sample 3 (nanocellulose fiber nanocomposites) exhibited a reinforcing effect at lower concentrations, unlike Samples 1 (spherical calcium carbonate nanocomposites) and 2 (nanotubular halloysite nanocomposites), making Sample 3 more promising for load-bearing implant components. However, yield stress was not significantly influenced by either filler concentration or its interaction with sample type. Although Sample 3 demonstrated improved reinforcement due to even dispersion and good matrix compatibility, this suggests its potential for applications where yield strength is crucial. Fracture toughness decreased with increasing nanofiller concentration, indicating an optimal range of 0.250 to 0.500 w.% for balancing stiffness, strength, and toughness. A significant decrease at 1.000 w.% across all fillers, particularly in Sample 3, suggests a limit to nanocellulose fiber reinforcement at high concentrations, which is critical for implant longevity. Dynamic mechanical vibration testing and microhardness measurements showed no statistically significant differences in *f_R_*_1_ and *E_IT_* for nanofiller concentration or sample type, but indentation creep showed a significant relationship between concentration and *C_IT_* (*p* = 0.007). Indentation hardness measurements revealed statistically significant differences for both concentration (*p* = 0.007) and sample type (*p* = 0.024). Finally, Shore D hardness generally increased with nanofiller concentration, with the highest increase observed at 1.000 w.%. Sample 3 exhibited the highest hardness, at 0.125 to 0.500 w.%, highlighting its potential to enhance the surface properties of biomedical composites for orthopedic implants and other mechanically resistant components. These findings suggest a tailored composite design, incorporating specific filler types and concentrations, to optimize long-term mechanical performance.

## Data Availability

The data that support the findings of this study are openly available in [Zenodo] at https://doi.org/10.5281/zenodo.14928686 (accessed on 25 March 2025) [32].

## Supplementary Materials

The following supporting information can be downloaded at: https://www.mdpi.com/article/10.3390/polym17070909/s1, Figure S1. Force vs. stroke dependences of the studied nanocomposite materials with 1.00 w.% filler concentration and neat ABS.
