# Supplementary material for: ABS Nanocomposites for Advanced Technical and Biomedical Applications
Source: Polymers (Basel). 2025 Mar 27;17(7):909. doi: 10.3390/polym17070909 (PMC11991112; doi:10.3390/polym17070909)
Supplement: Supplementary file 1 [file polymers-17-00909-s001.zip › polymers-3531075-supplementary.pdf]

**Supplementary Materials**

# ABS Nanocomposites for Advanced Technical and Biomedical Applications

**Lubomír Lapčík [1,2,*], Martin Vašina [2,3], Yousef Murtaja [2], Harun Sepetcioglu [4], Barbora Lapčíková [1,2], Martin Ovsík [2], Michal Staněk [2], İdris Karagöz [5] and Apurva Shahaji Vadanagekar [1]**

[1] Department of Physical Chemistry, Faculty of Science, Palacky University, 17. Listopadu 12, 771 46 Olomouc, Czech Republic; barbora.lapcikova@upol.cz (B.L.); apurva.vadanagekar01@upol.cz (A.S.V.)
[2] Faculty of Technology, Tomas Bata University in Zlin, Vavreckova 5669, 760 01 Zlin, Czech Republic; martin.vasina@vsb.cz (M.V.); murtaja@utb.cz (Y.M.); ovsik@utb.cz (M.O.); stanek@utb.cz (M.S.)
[3] Department of Hydromechanics, Faculty of Mechanical Engineering, VŠB—Technical University of Ostrava, and Hydraulic Equipment, 17. Listopadu 15/2172, 708 33 Ostrava-Poruba, Czech Republic
[4] Department of Metallurgy and Materials Engineering, Technology Faculty, Selçuk University, 42075 Konya, Türkiye; harunsepet@selcuk.edu.tr
[5] Department of Polymer Materials Engineering, Faculty of Engineering, Yalova University, 77200 Yalova, Türkiye; idris.karagoz@yalova.edu.tr
* Correspondence: lubomir.lapcik@upol.cz

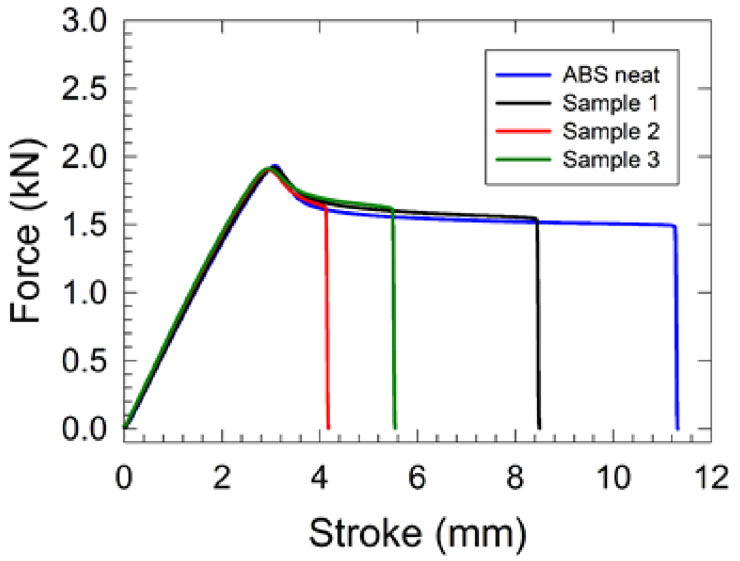


Figure S1. Force vs. stroke dependences of the studied nanocomposite materials with 1.00 w.% filler concentration and neat ABS.
